# Active Video Games to Improve Behavioral Intentions and Cognitive Function in Patients With Schizophrenia: Randomized Controlled Trial

**DOI:** 10.2196/69116

**Published:** 2025-10-01

**Authors:** Huan-Hwa Chen, Ching-Ching Lin, Man-Ling Yu, Hsiu-Lan Wu, Hui-Chu Shen, Hsiu-Fen Hsieh

**Affiliations:** 1 Department of Nursing Chung Hwa University of Medical Technology Tainan Taiwan; 2 Department of Nursing, Taipei Veterans General Hospital, Yuanshan Branch Yilan Taiwan; 3 School of Nursing, Kaohsiung Medical University Kaohsiung Taiwan; 4 Department of Medical Research, Kaohsiung Medical University Hospital, Kaohsiung Medical University Kaohsiung Taiwan

**Keywords:** schizophrenia, behavioral intention, cognitive function, active video games

## Abstract

**Background:**

Schizophrenia is a severe mental illness that affects the cognitive, social, and daily functions of patients. Physical activity has been found to be important for maintaining these functions in patients with schizophrenia, but many lack the motivation to participate in physical activities.

**Objective:**

This study aimed to explore the efficacy of active video games (AVGs) on the behavioral intention and cognitive function of patients with schizophrenia.

**Methods:**

In this experimental study, 103 participants were recruited from 2 medical centers. All participants were randomly assigned to the experimental or control group, and 82 participants (n=41, 50% in the experimental group and n=41, 50% in the control group) completed all the processes of our protocol. The experimental group was provided with AVGs for 30 minutes twice per week for 6 weeks. The Mini Mental State Examination and a behavioral intention questionnaire were administered before and after playing the AVGs. Data were collected between April 2021 and January 2022. Generalized estimating equations and 2-tailed paired *t* tests were used for data analysis.

**Results:**

The experimental group showed significant improvements in behavioral intention to participate in AVGs compared with the control group at both T1 (β=4.88; *P*=.009) and T2 (β=4.24; *P*=.04). In addition, the experimental group experienced significant improvements in orientation (T2: β=0.66; *P*=.04) and language (T2: β=0.28; *P*=.03) among cognitive functions compared to the control group. In contrast, there was no significant change in these variables in the control group.

**Conclusions:**

Playing AVGs can effectively enhance the behavioral intention of patients with schizophrenia to participate in physical activity and exercise and significantly improve their orientation and language. AVGs are inexpensive and easily operated tools for people with mental or physical disabilities.

**Trial Registration:**

ClinicalTrials.gov NCT05933356; https://clinicaltrials.gov/study/NCT05933356

## Introduction

### Background

Schizophrenia can lead to high functional disability and is associated with a heavy economic burden worldwide. The World Health Organization lists it as one of the top 10 conditions in terms of the global burden of disease [1,2]. Schizophrenia is a disease characterized by impairments in cognitive functions. Positive symptoms of this disorder, such as delusions and hallucinations, respond relatively well to antipsychotic medication, whereas the management of negative symptoms remains a clinical challenge [3,4]. Negative symptoms are associated with severe deterioration in cognition and global functioning [5,6]. Patients with negative symptoms often exhibit weak motivation for activities, which accelerates the degradation of cognitive functions. Schizophrenia is a condition that inherently leads to impaired cognitive function, compounded by patients’ low motivation to engage in activities, which further accelerates cognitive decline. A study conducted in Singapore evaluated the utility of the Mini Mental State Examination (MMSE) among long-term hospitalized patients diagnosed with schizophrenia or schizoaffective disorder. The findings indicated that MMSE scores were significantly influenced by sociodemographic and clinical variables, including age, educational attainment, and duration of illness [7]. A study conducted in the United States assessed partially remitted outpatients attending a depot antipsychotic clinic or a clozapine clinic (N=272) using the MMSE. The findings indicated that the cognitive gap did not appear to widen significantly with increasing age. The most commonly observed deficits among patients with poorer performance were in memory, attention, and constructional tasks. The MMSE was found to be easy to administer and applicable for use among outpatient populations with schizophrenia [8].

Exercise not only improves physical function but also serves as a crucial factor in enhancing cognitive function. Multiple factors hinder the efficacy of treating patients with schizophrenia, including limited insight, poor adherence, treatment resistance [9], antipsychotic-related side effects [10], and cultural factors [11]*.* Therefore, new treatment options are required to promote recovery and optimize rehabilitation [12]. Many studies have indicated that exercise can be an additional option for the prevention and treatment of schizophrenia [4,13-15] and it can improve patients’ positive and negative symptoms, quality of life, and cognitive function [13,16]. In addition, exercise enhances physical well-being and decreases potential somatic comorbidities such as metabolic syndrome [3] and cardiovascular diseases in patients with schizophrenia [3]. A systematic review indicated that exercise significantly improved patients’ working memory, social cognition, and attention and vigilance [17].

A previous systematic review identified exercise interventions as one of the most effective strategies for improving mental health. These interventions typically lasted for 1 to 3 months, with a frequency of 2 to 3 sessions per week and most sessions lasting between 30 and 60 minutes [18]. Exercise not only increases cerebral blood flow—providing the brain with more oxygen and nutrients—but also reduces neuronal apoptosis, promotes neurogenesis and brain plasticity, and enhances cognitive reserve. A previous study systematically reviewed the relationship among exercise, brain-derived neurotrophic factor, neural plasticity, and cognitive function. The results indicated that exercise can elevate brain-derived neurotrophic factor levels in the brain, thereby promoting neural plasticity and enhancing cognitive function [19].

Most patients with schizophrenia lack motivation for physical activities such as exercise and social interaction owing to various factors, including the effects of negative symptoms [3,4,18,19]. However, in recent decades, they have been recognized as interested in modern technology-assisted activities [20], and such activities are a possible method of rehabilitation. For example, video games, active video games (AVGs) [21-24], and virtual reality [25] have been documented as novel interventions for cognitive training and for alleviating psychotic symptoms in patients with schizophrenia [24,26,27]. A systematic review also found a positive relationship between video games and cognitive functions such as memory [28-30], social cognition [31], executive function [32], attention [33], and reaction time [34]. It has been suggested that playing video games can have cognitive, motivational, emotional, and social benefits [23] with a lower cost than those of other cognitive interventions [35].

With the rapidly changing technologies in today’s world, AVGs not only are fun but can also serve as exercise tools, making it more convenient for people to exercise. AVGs refer to exercises that use video games to make exercise more fun [36]. A previous systematic review focused on interactive motion-sensing games that required players to engage in physical movements such as waving, jumping, kicking, or full-body motion to control internet-based characters or complete in-game tasks. These games integrate entertainment with physical activity, breaking away from the traditional sedentary gaming model [37]. AVGs are considered capable of enhancing an individual’s behavioral intention to engage in these activities by fostering imagination, control, challenge, and curiosity. Engagement in exercise can increase either through consistently finding satisfaction in familiar activities or by trying new ones to sustain interest and motivation. [38]. One study reported that >90% of the participants were satisfied with playing AVGs, and most participants (76.9%) agreed that this intervention promotes healthier lifestyles and is an acceptable alternative way to perform physical activity [39]. AVGs significantly improve cognitive domains, including processing speed, attention and vigilance, working memory, verbal learning, visual learning, reasoning and problem-solving [40], social cognition, social functioning, and motivation [24] and also reduce cognitive impairments and psychological stress [41]. A systematic review indicated that AVGs were globally beneficial for patients with schizophrenia, particularly with regard to their cognitive function [42].

In summary, AVGs are accessible and easy to operate for patients with disabilities [43,44], and many studies indicate that interventions integrating gameplay with physical exercise can enhance motivation for device-assisted activities; alleviate residual symptoms; and improve cognitive, social, and occupational functioning, thereby contributing to an improved quality of life among patients with schizophrenia. Nevertheless, despite the growing global emphasis on digital health interventions, studies specifically investigating the impact of AVGs on this population in Taiwan remain scarce. This highlights a critical need to develop innovative, accessible, and engaging strategies to facilitate recovery and rehabilitation in patients living with schizophrenia. Furthermore, addressing cognitive deficits—a core and profoundly disabling characteristic of the disorder—is imperative due to their significant influence on daily functioning and overall well-being [19].

### Objectives

This study aimed to explore the role of AVGs in influencing the behavioral intention of patients with schizophrenia to participate in novel device-assisted activities and examine their efficacy in improving cognitive function.

We hypothesized that the experimental group would show greater improvement in cognitive function than the control group at each assessment point (3 and 6 weeks) during the intervention. In addition, we expected that playing AVGs would increase the experimental group’s behavioral intention to continue engaging with AVGs after the intervention.

## Methods

### Study Design

The protocol was registered with ClinicalTrials.gov (NCT05933356) at the time of submission. We used an experimental design with random sampling, treating each day care center as a unit. Patients within these centers were assigned to either the experimental or control group using a random number generator (Microsoft Excel). This study was single blinded, with the research assistants being the blinded party. All participants received usual care, whereas those in the experimental group additionally engaged with AVGs over the 6-week study period.

### Participants and Setting

This was an experimental study with participants recruited from day care centers from 2 hospitals in southern Taiwan. The MMSE and a behavioral intention questionnaire were administered before and after playing the AVGs.

The sample size was calculated using G*Power (version 3.1.1) for the *F*_1_ test and its repeated measures with an effect size of 0.25, a significance level of .05, a power (1–β) of 0.80, and an estimated minimum sample size of 78 [45]. A total of 103 patients with schizophrenia participated in our study, and 82 (79.6%) completed all intervention processes and questionnaires. Before data collection, the principal investigator explained the purpose and process to the nurses in each ward and invited them to participate in the study.

The inclusion criteria for patients were as follows: (1) diagnosis of schizophrenia according to the *Diagnostic and Statistical Manual of Mental Disorders, Fifth Edition* [46]; (2) age of 20 to 65 years; (3) ability to read and understand traditional Chinese; and (4) current admission to a psychiatric day care center. The exclusion criteria were as follows: (1) patients with unstable mental conditions, such as delusions or hallucinations; and (2) patients with an intellectual disability or severe cognitive function impairment (MMSE score of <14) [47].

### Study Protocol

#### Control Group

The control group only received scheduled activities in the day care center as usual without any intervention.

#### Experimental Group

The experimental group received routine care and participated in AVGs from Nintendo Ltd played for 30 minutes twice a week over 6 weeks. At the start, participants were instructed to play games designed to enhance attention, spatial intelligence, and enjoyment in a safe, spacious environment. Each patient acted as an independent player, engaging in fair competition that fostered intrinsic motivation and enhanced the ability to observe others’ behaviors.

Participants were guided in playing the AVGs by the research team. Each session lasted approximately 30 minutes. Measurements were recorded at baseline, week 3, and week 6, designated as T0, T1, and T2, respectively.

### Data Collection

Each participant was required to complete a demographic characteristic and behavioral intention questionnaire, and their cognitive functions were assessed using the MMSE. The principal investigator initially collected participants’ demographic information through structured interviews. To ensure the accuracy and completeness of the data, the collected information was subsequently verified in collaboration with nurses at the day care center. Data were collected between April 2021 and September 2022.

### Instruments

#### The Mario Party Series (Nintendo Ltd)

*Mario Party* is a party video game series featuring characters from the Mario franchise where up to 4 local players or computer-controlled characters compete in a board game interspersed with mini games. The games are currently developed and published by Nintendo. The *Mario Party* games feature at least 80 mini games across various types. Four-player games are typically free-for-all, where players compete individually. In other mini games, players form teams to cooperate and win. We used the Joy-Cons of the Nintendo Switch to control the game. Joy-Cons consist of 2 individual units, each containing an analog stick and a set of buttons. They can be used while attached to the main Nintendo Switch console unit or detached for wireless use. When detached, a pair of Joy-Cons can be used by a single player or split between 2 players as individual controllers. Each Joy-Con includes an accelerometer and gyroscope for motion tracking (Table 1).

**Table 1:** Name and description of each minigame.

Minigame	Image	Goals	Content
Sizzling Stakes	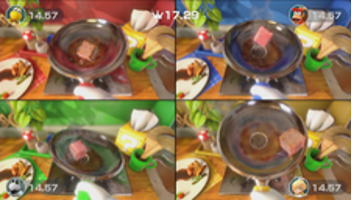	Enhance the patient’s orientation	Shake the controller to control the pan. All 6 sides of the beef must be pan-seared to secure a win. Gameplay: 4 individuals play the game at the same time, independently but concurrently.
Snack Attack	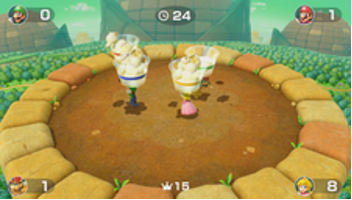	Enhance the patient’s orientation	Catch popcorn in a cup by controlling the remote button; 1point for each caught piece of popcorn. Gameplay: 4 individuals play the game at the same time, independently but concurrently.
Candy Shakedown	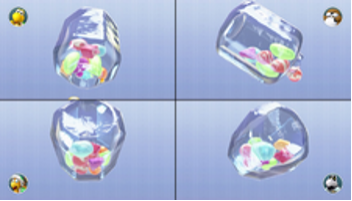	Enhance the patient’s pace and orientation	Pour out all the candies from the jar by shaking the controller. The player with the fastest time is the winner. Gameplay: 4 individuals play the game at the same time, independently but concurrently.
Precision Gardening	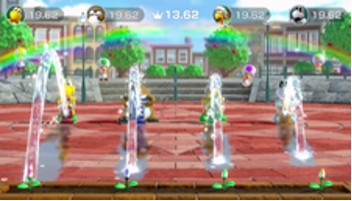	Enhance the patient’s hand strength and reasoning	Shake both controllers in a downward direction to water the flowers; the flower blooms as fast as the speed of shaking. The fastest player wins the game. Gameplay: 4 individuals play the game at the same time, independently but concurrently.
Pie Hard	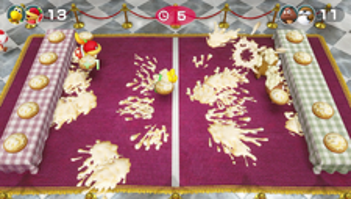	Enhance the patient’s communication, attention and reasoning	Throw pies to the opposite side by clicking the button; a point is earned when a pie hits a competitor. Gameplay: 4 individuals play the game at the same time, independently but concurrently.
Tall Order	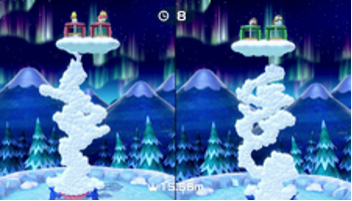	Enhance the patient’s orientation	Use the controller to catch as ice in the bowl as possible. Gameplay: 4 individuals play the game at the same time, independently but concurrently.
Rowboat Uprising	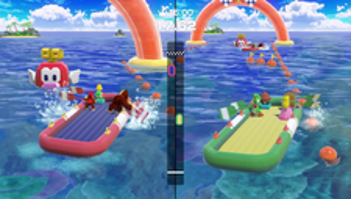	Enhance the patient’s communication, attention and reasoning	The faster the controller is shaken, the less time is needed to sail to the goal. Players play the game in teams of two.
Net Worth	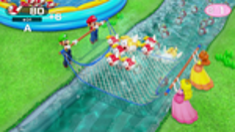	Enhance the patient’s communication, attention and reasoning	Two players are in one team. The arms are lifted up together to catch fish while they swim through the net. Players play the game in teams of two.
Strike It Rich	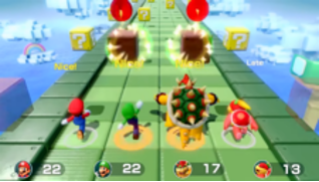	Enhance the patient’s attention and reasoning	The hands are lifted up when the character encounters a brick in the race. One point is won when the character hit a brick successfully. Gameplay: 4 individuals play the game at the same time, independently but concurrently.
Take a Stab	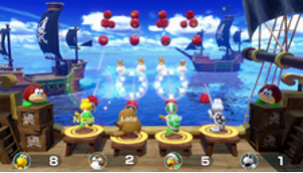	Enhance the patient’s attention and reasoning	The arms are stretched forward when the fruit is close to the sword. Player can use beats shown on screen for timing. Gameplay: 4 individuals play the game at the same time, independently but concurrently.
Wiped Out	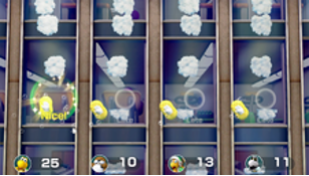	Enhance the patient’s executive function and reasoning	Wipe soap from windows by waving the hands in a circular motion. A point is earned each time a soap bubble is wiped off. Gameplay: 4 individuals play the game at the same time, independently but concurrently.
Clearing the Table	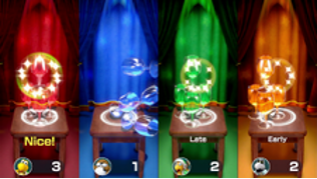	Enhance the patient’s attention and reasoning	Pull the tablecloth quickly when enough cups are placed on the table by shaking the hands. One success leads to 1 point earned. Gameplay: 4 players play together simultaneously, each controlling their own character.

#### Behavioral Intention Questionnaire Instrument

We measured participants’ behavioral intention questionnaire to play AVGs using a self-developed questionnaire that was formulated with input from 4 experts and consisted of 4 items. Each item was rated on a 5-point Likert scale to assess the level of agreement with each statement, ranging from 0 (*strongly disagree*) to 4 (*strongly agree*). The questionnaire contained the following questions: “In your opinion, will you play and try different AVGs in the future? Do you think that AVGs can make you relaxed or vigorous?” “In your opinion, will you be interested in trying different types of AVGs?” “Do you think that AVGs can make you vigorous?” The content validity index of the questionnaire was 1.00, indicating excellent content validity. The total score for the 4 items ranged from 0 to 16. Higher scores indicated a stronger intention to play AVGs. The test-retest (2 weeks) intraclass correlation coefficient was 0.88. The Cronbach α for behavioral intention was 0.93 in this study.

#### MMSE Instrument

Cognitive tests were conducted using the MMSE [47], which is one of the best-known and most widely used instruments for assessing cognitive function in clinical practice worldwide. The MMSE comprises 11 questions covering 6 cognitive domains: orientation, attention and calculation, memory, language, reasoning and problem-solving, and executive function [48]. The total score ranges from 0 to 30, with higher scores indicating better cognitive function. The MMSE sensitivity was good (0.83, 95% CI 0.62-0.95), and the specificity was very good (0.99, 95% CI 0.92-0.99). The Cronbach α was 0.78 [49]. The behavioral intention questionnaire and MMSE questionnaires are available in Multimedia Appendix 1.

### Data Analysis

Descriptive analyses using chi-square and independent-sample *t* tests were conducted using the SPSS software (version 21.0; IBM Corp). Descriptive analysis was based on participants’ demographic characteristics, such as age, gender, educational level, marital status, and religious beliefs. After controlling for the confounding factor of age, a generalized estimating equation was used to assess changes in behavioral intention and cognitive functions (including orientation, memory, attention and calculation, language, reasoning and problem-solving, and executive function) from baseline to the end of the intervention in the 2 groups. All tests were 2-tailed, with a significance level set at *P*<.05.

### Ethical Considerations

This study was approved by the institutional review boards of Kaohsiung Medical University Chung-Ho Memorial Hospital (KMUHIRB-SV (I)–20200105) and Taipei Veterans General Hospital (2023-07-002A). Written informed consent was obtained from all participants, and they were assured of data confidentiality and anonymity and that they could withdraw from the study at any time without providing a reason. The principal investigator visited each psychiatric day care center, explained the study’s purpose and process to all participants, and inquired regarding their willingness to take part. Each participant was offered TWD $400 (US $13-$14) as compensation for their taking part.

## Results

### Baseline Data

**Figure 1:**
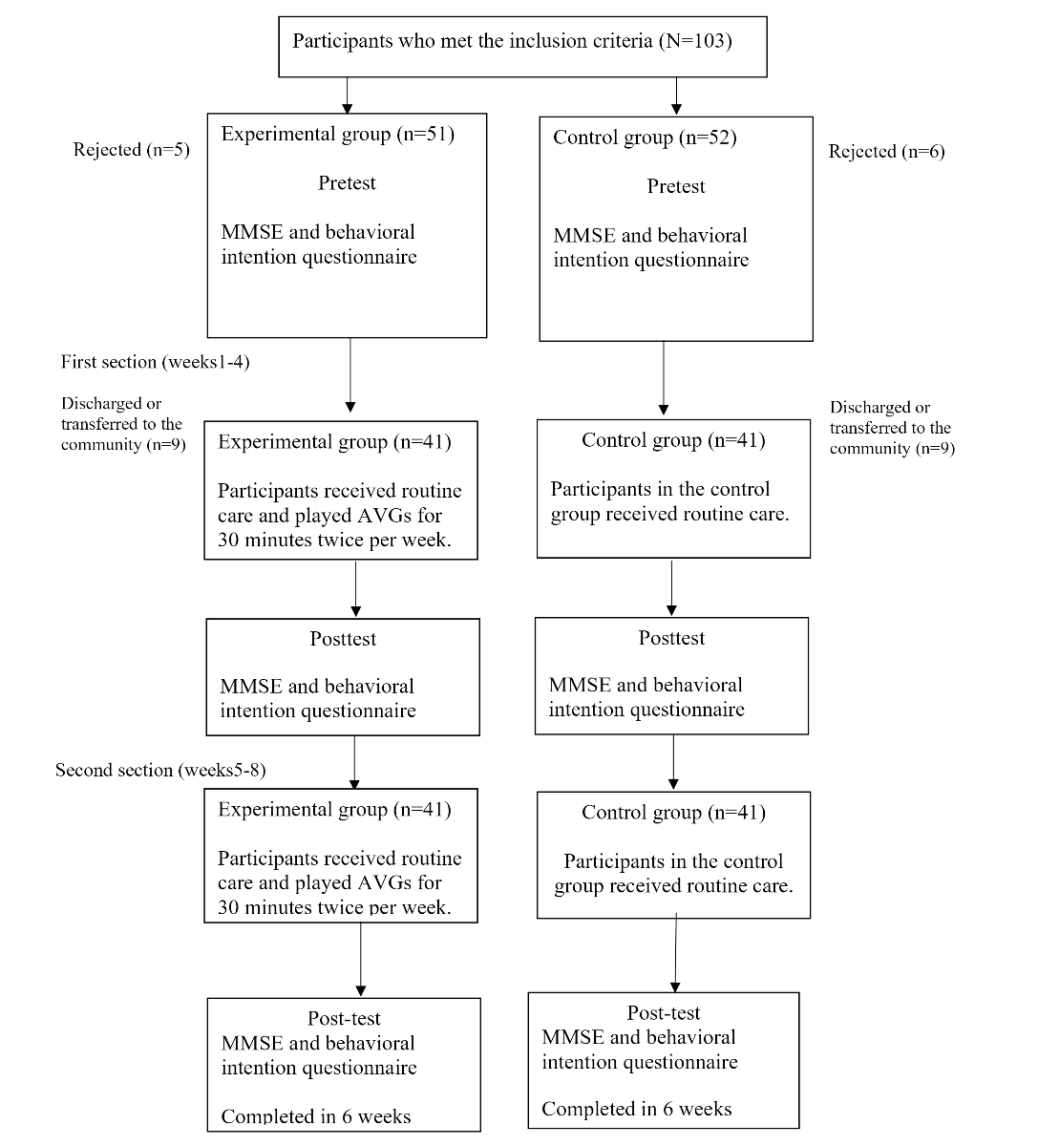
Participant flowchart. AVG: active video game; BIQ: behavioral intention questionnaire; MMSE: Mini Mental State Examination.

A total of 82 participants met the inclusion criteria and completed all the processes of our protocol. The participants were divided into the experimental and control groups (Figure 1), and we compared demographic data between these groups. The independent-sample 2-tailed *t* test and chi-square test results showed no statistically significant differences between the 2 groups (Table 2).

**Table 2:** Demographic characteristics and baseline outcomes.

Characteristic			Experimental group (n=41)		Control group (n=41)		Chi-square (*df*)			*t* test (*df*)			*P* value	
Age (y), mean (SD)			43.61 (12.2)		47.95 (10.6)		—^a^			1.72 (80)			.09	
**Sex, n (%)**								1.00 (1)			—			.59
	Male	19 (46)		19 (46)										
	Female	22 (54)		22 (54)										
**Hypertension, n (%)**								0.746 (1)			—			.50
	No	35 (85)		36 (88)										
	Yes	6 (15)		5 (12)										
**Diabetes mellitus, n (%)**								0.312 (1)			—			.16
	No	34 (83)		38 (93)										
	Yes	7 (17)		3 (7)										
**Level of education, n (%)**								3.57 (5)			—			.61
	Junior high school	6 (15)		6 (15)										
	Senior high school	25 (61)		22 (54)										
	College	10 (24)		11 (27)										
	Higher than college	0 (0)		2 (5)										
**Marital status, n (%)**								3.20 (3)			—			.36
	Single	34 (83)		32 (78)										
	Divorced	3 (7)		6 (15)										
	Married	4 (10)		3 (7)										
**Religious beliefs, n (%)**								3.13 (1)			—			.08
	Yes	31 (76)		25 (61)										
	No	10 (24)		16 (39)										
Onset age (y), mean (SD)			27.40 (12.3)		24.74 (8.6)		—			−1.09 (72)			.28	
BMI (kg/m^2^), mean (SD)			26.40 (5.4)		25.58 (6.8)		—			−0.59 (16)			.55	
**Living status, n (%)**								6.44 (1)			—			.17
	Living alone	6 (15)		9 (22)										
	Living with their family or friends	35 (85)		32 (78)										
MMSE^b^ score (0-30), mean (SD)			26.76 (3.2)		25.34 (4.2)		—			−1.720 (81)			.09	
Behavioral intention score (0-16), mean (SD)			13.75 (3.8)		13.66 (3.3)		—			−0.124 (81)			.90	

^a^Not applicable.

^b^MMSE: Mini Mental State Examination.

### Changes in Cognitive Function and Behavioral Intention From Baseline to the End of the Intervention

We controlled the age and educational level as the MMSE scores were related to both of these factors. We used generalized estimating equations to analyze changes in participants’ cognitive functions and found significant improvements in the cognitive domains of orientation (T2: β=0.66; *P*=.04) and language (T2: β=0.28; *P*=.03), as well as in behavioral intention (T1: β=4.88 and *P*=.009; T2: β=4.24; *P*=.04), following the intervention with AVGs compared to baseline (T0) in the experimental group (Table 3).

**Table 3:** Changes in cognitive function from baseline to the end of the intervention.

Parameter	EG^a^ vs CG^b^			T2^c^ vs T0^d^			T1^e^ vs T0			EG × T2 vs CG × T2			EG × T1 vs CG × T1	
	β (SE)	*P* value	β (SE)		*P* value	β (SE)		*P* value	β (SE)		*P* value	β (SE)		*P* value
MMSE^f^	0.29 (2.90)	.92	−0.42 (1.53)		.79	0.08 (1.44)		.95	−0.13 (0.67)		.85	0.36 (0.60)		.55
Orientation	0.44 (1.43)	.56	0.57 (0.65)		.35	0.66 (0.70)		.40	1.90 (0.33)		.04^g^	1.76 (0.32)		.08
AC^h^	1.42 (1.27)	.26	−0.79 (0.88)		.37	0.90 (0.70)		.20	−0.15 (0.45)		.73	−0.45 (0.34)		.18
Language	1.09 (0.39)	.89	1.05 (0.30)		.11	0.98 (0.28)		.29	1.24 (0.13)		.03^g^	1.22 (0.13)		.09
RPS^i^	−0.18 (0.39)	.65	−0.22 (0.38)		.56	−0.52 (0.32)		.10	0.07 (0.16)		.66	0.21 (0.14)		.13
Memory	−0.34 (0.51)	.50	0.06 (0.49)		.90	0.44 (0.40)		.28	−0.01 (0.20)		.94	0.19 (0.20)		.35
EF^j^	−0.04 (0.24)	.87	−0.26 (0.27)		.33	−0.38 (0.25)		.12	0.17 (0.13)		.20	0.09 (0.11)		.43
Behavioral intention	69.88 (2.61)	.10	0.82 (0.46)		.67	1.16 (0.38)		.70	4.24 (0.69)		.04^g^	4.88 (0.60)		.009^k^

^a^EG: experimental group.

^b^CG: control group.

^c^T2: 6-week time point.

^d^T0: baseline.

^e^T1: 3-week time point.

^f^MMSE: Mini Mental State Examination.

^g^*P*<.05.

^h^AC: attention and calculation.

^i^RPS: reasoning and problem-solving.

^j^EF: executive function.

^k^*P*<.01.

## Discussion

### Principal Findings

The experimental group had significantly higher behavioral intention to participate in AVGs and showed significant improvements in the cognitive domains of orientation and language compared to the control group. These findings indicate that the intervention not only enhanced motivation among patients with schizophrenia to participate in AVG-based activities but also led to measurable cognitive gains. Taken together, the results suggest that AVG interventions are both feasible and potentially effective in simultaneously addressing cognitive deficits and motivational challenges in this population.

### Comparison to Previous Work

#### Behavioral Intention

Our results indicated a significant enhancement in the behavioral intention of patients with schizophrenia to participate in AVGs after the intervention. This result is similar to the results of previous studies [50] that have indicated that interventions incorporating multisensory stimulation can enhance the motivation of patients with schizophrenia to engage in device-assisted activities [51,52]. A plausible explanation for the observed improvements is that AVGs simultaneously activate multiple sensory modalities, including visual, auditory, and tactile systems. This multisensory stimulation fosters a more immersive and engaging experience, which may contribute to increased participation. Such experiences can help stimulate cognitive processing and enhance attention, memory, and motor skills. In addition, the dynamic and interactive nature of AVGs can create an environment that promotes relaxation, reduces stress, and fosters a sense of accomplishment.

The combination of enjoyable gameplay and positive emotional responses such as satisfaction and pleasure could further contribute to improved cognitive function by increasing motivation and engagement during the intervention. Due to these features of novel device-assisted activities, it is feasible to use AVGs as a tool to change the behavioral intention for physical activity or exercise in patients with schizophrenia.

#### Cognitive Function: Significant Improvements in the Language and Orientation Domains

We also found that language improvement was consistent with that found in previous research [40], which reports enhanced verbal abilities following AVG engagement. One plausible explanation for this outcome is that AVGs activate the brain’s reward system, which may, in turn, facilitate verbal learning and memory consolidation in patients with schizophrenia [53]. These gains were reflected in both expressive and receptive capacities. Specifically, participants demonstrated enhanced abilities in verbal communication, including word retrieval and fluency, as well as improved comprehension of spoken language. Such improvements are particularly meaningful for patients with schizophrenia as language dysfunction is closely associated with impaired social interaction, reduced functional capacity, and poor treatment adherence. Thus, the observed enhancements underscore the potential of structured, technology-assisted interventions to promote not only cognitive processing but also communicative competence, which is an essential element in real-world rehabilitation and recovery.

In addition to gains in language, our study revealed significant enhancement in orientation. Interestingly, such outcomes have been less commonly reported in previous AVG intervention studies, suggesting a novel contribution of our findings. Most previous research has focused on improvements in general cognitive functioning, executive function, or memory, with limited emphasis on orientation as a distinct outcome [54]. The observed improvement in orientation may be attributed to the specific design characteristics of the games used—namely, the *Mario Party* series. These games incorporate spatial and directional challenges that require players to navigate complex virtual environments, follow sequential directions, and make decisions based on their spatial understanding. Such tasks likely stimulate cognitive processes related to spatial awareness, planning, and environmental integration, all of which are integral to orientation. Therefore, our findings expand the scope of known cognitive benefits associated with AVG interventions by highlighting their potential to enhance orientation through immersive spatial gameplay.

#### Cognitive Function: No Significant Changes in Attention and Calculation, Reasoning and Problem-Solving, Memory, and Executive Function

One plausible explanation for this finding pertains to the selection of the cognitive assessment instrument. In this study, cognitive function was measured using the MMSE, a widely used screening tool in clinical settings. While the MMSE offers the advantage of brevity and ease of administration, it is well recognized for its limited sensitivity in detecting deficits across several higher-order cognitive domains, including attention and calculation, reasoning and problem-solving, memory, and executive function [47]. These limitations may have constrained the detection of subtle but meaningful changes resulting from the intervention and may account for the absence of significant findings within these domains in our sample. Given that these cognitive functions are critical to daily functioning and are often impaired in neuropsychiatric populations such as patients with schizophrenia, reliance on the MMSE alone may underestimate the true extent of cognitive deficits [55]. Consequently, we recommend that future research incorporate more sensitive and domain-specific assessment instruments such as the Montreal Cognitive Assessment, which has demonstrated superior efficacy in detecting subtle cognitive impairments, particularly in patients with schizophrenia [56]. Using such tools will likely yield a more comprehensive and accurate profile of cognitive dysfunction, thereby informing targeted interventions and improving clinical outcomes.

Previous studies have implemented AVG interventions using gaming consoles such as the Nintendo Wii and Xbox Kinect primarily targeting physical activity or cognitive stimulation in diverse clinical populations. A systematic review reported that 44% of AVG-based interventions used the Nintendo Wii console and 36% used the Xbox Kinect console, reflecting the widespread adoption of these platforms in therapeutic settings [57]. Diener et al [53] further noted that, although these interventions often shared similar durations and frequencies, critical procedural details such as session content, gameplay structure, and supervision level were frequently underreported. This lack of detail limits both replicability and generalizability [53]. In contrast, this study adopted a clearly defined and systematically implemented 6-week AVG intervention protocol. However, it is possible that the intervention duration and exposure frequency were insufficient to elicit more robust improvements in higher-order cognitive domains. Complex cognitive abilities such as memory and executive function typically require more intensive and sustained engagement to show measurable gains [58].

### Implications for Rehabilitation and Clinical Practice

In patients with schizophrenia, cognitive impairment and motivational deficits are well-documented barriers to rehabilitation [59], and our results suggest that AVGs may help address both. Compared to traditional rehabilitation methods, which often require extensive professional oversight and may lack engaging elements, AVGs offer an interactive, motivating, and low-barrier alternative [60,61]. Traditional therapeutic activities in psychiatric day care settings, such as arts and crafts, group discussions, or paper-based cognitive tasks, while beneficial, are often passive, repetitive, or fail to sufficiently motivate patients with negative symptoms or attentional difficulties [62]. In contrast, AVGs offer a dynamic and patient-centered alternative with several distinct advantages. AVGs are interactive, provide real-time feedback, and present task-oriented challenges within immersive environments. These features are particularly effective in sustaining attention and promoting active engagement. Importantly, AVGs can be administered with minimal supervision and infrastructural demands, making them well suited for integration into community-based programs and other low-resource contexts [58-62].

Our findings support the potential of AVGs to address both cognitive and motivational deficits. Participants in the intervention group not only showed significant improvements in the cognitive domains of orientation and language but also demonstrated increased behavioral intention to engage with AVGs. The structured yet enjoyable gameplay likely fostered a sense of mastery and self-efficacy, which are key factors in enhancing intrinsic motivation. This is particularly relevant for patients with schizophrenia, who often struggle with motivation and social withdrawal.

While overall cognitive function did not show significant improvement, we observed notable gains in the domains of orientation and language. These domains are often relatively preserved and more responsive to interventions in patients with mild cognitive impairment or certain psychiatric conditions [63,64]. In addition, AVGs can significantly impact behavioral intention by motivating patients to engage in physical activity. For patients with schizophrenia, who often experience difficulties with motivation and social engagement, the interactive nature of AVGs provides a compelling, enjoyable way to encourage participation in exercise. The games’ structured yet fun activities help foster a sense of achievement, which can translate into more consistent engagement in physical activity. Taken together, these findings highlight the feasibility and clinical utility of AVGs as a scalable, low-barrier intervention for improving select cognitive domains and enhancing motivation among patients with schizophrenia. Their affordability, accessibility, and adaptability to various patient needs position them as promising adjuncts to traditional rehabilitation strategies in resource-limited or community-based psychiatric programs.

### Future Directions

First, it is unclear whether the cognitive and behavioral gains associated with AVGs persist over time or depend on continued engagement. Second, future studies should explore how AVGs can be integrated into comprehensive, multidisciplinary care models. Personalizing AVG content according to patients’ cognitive status, physical capabilities, or symptom severity may further improve intervention efficacy. Third, conducting subgroup analyses across age groups, illness stages, and care settings could inform more personalized and scalable implementation strategies.

### Limitations

Our study has several limitations. First, we provided a relatively lower intensity and shorter duration of the intervention for patients with schizophrenia, which may limit the benefits of AVGs observed in our participants. Second, our sample size was sufficient but relatively small, which may limit the generalizability of the findings to the broader population. Further studies with a larger sample size, higher intervention intensity, and longer duration, along with long-term follow-up, are needed to overcome these limitations. Finally, we used the MMSE to assess cognitive function. However, the MMSE has limited sensitivity to executive function and working memory, which may explain the lack of significant findings in these domains. For future research, we recommend using the Montreal Cognitive Assessment as it is more effective in detecting cognitive impairments, particularly in patients with schizophrenia or mild cognitive impairment.

### Conclusions

This study contributes to the growing body of evidence supporting AVGs as effective and engaging interventions for improving orientation, language, and behavioral motivation in patients with schizophrenia. Our structured 6-week program yielded meaningful improvements in these domains, which are often difficult to impact through traditional approaches. Although preliminary, the results highlight the potential of well-designed AVG interventions as scalable, low-barrier tools for psychiatric rehabilitation. Unlike conventional therapies that frequently rely on specialized resources or highly trained professionals, AVGs offer a flexible alternative suitable for diverse settings, including psychiatric wards, outpatient clinics, and community centers.

## Appendix

Multimedia Appendix 1 The questionnaire on behavioral intention and the Mini-Mental State Examination in both the Chinese (original) and English versions.

Multimedia Appendix 2 CONSORT-eHEALTH checklist (V 1.6.1).

## Abbreviations

AVG: active video game
MMSE: Mini Mental State Examination

## Data Availability

The datasets generated or analyzed during this study are not publicly available due to the inclusion of sensitive and confidential information but are available from the corresponding author on reasonable request.
