# Supplementary material for: Active Video Games to Improve Behavioral Intentions and Cognitive Function in Patients With Schizophrenia: Randomized Controlled Trial
Source: JMIR Serious Games. 2025 Oct 1;13:e69116. doi: 10.2196/69116 (PMC12530152; doi:10.2196/69116)
Supplement: Multimedia Appendix 1 [file games_v13i1e69116_app1.pdf]

# 行為意向

| | |
|---|---|
| 1、未來您會願意參與體感式運動(遊戲機): | □非常不願意 □不願意 □普通 □願意 □非常願意 |
| 2、您會想要嘗試不同的體感運動(遊戲機)類型: | □非常不想要 □不想要 □普通 □想要 □非常想要 |
| 3、對您來說體感式運動(遊戲機)，可以讓您達到心情放鬆: | □非常不同意 □不同意 □普通 □同意 □非常同意 |
| 4、對您來說體感式運動(遊戲機)，可以讓您感到有活力： | □非常不同意 □不同意 □普通 □同意 □非常同意 |

認知功能評估量表

## 簡易心智量表（Mini-Mental State Examination；MMSE）

於 1975 年 Folstein 及 Mc Huge 所制定，評估項目包括**定向感、注意力、記憶力、語言、口語理解及行為能力、建構力**等項目，評估過程無時間限制，滿分是 30 分，分數越高表示認知功能越好，答對一項給一分，總分若低於 24 分表示個案有輕度認知功能障礙，若低於 16 分則表示有重度認知功能障礙。目前被廣泛使用。

全民健康保險規定診斷為阿茲海默氏症病患藥品給付為 **MMSE** 10~26 分。

| 滿分 30 分 | **國中以上教育** | 國小 | 未受教育 |
|---|---|---|---|
| | **＜24 分 輕度認知功能缺失**<br>**＜16 分 重度認知功能缺失** | ＜21 分<br>認知功能異常 | ＜16 分<br>認知功能異常 |

**簡易心智/認知狀態量表(MMSE)**

| 項目 | 最高分 | 分數 | 評分項目 |
|---|---|---|---|
| 一、定向感 (10) | 5<br>5 | (　)<br>(　) | 1.時間（5）：幾年？幾月？幾日？星期幾？什麼季節？<br>2 地方（5）：地方：縣/市？醫院？病房？床號？樓層？ |
| 二、注意力及計算能力(8) | 3 | (　) | 1.訊息登錄（3）：說出三項名詞(例如：房子、汽車、蘋果)：一秒中說一項，說完之後，要求說出這三項名詞，說對一項給一分，請個案記住，等一下會再請他說出這三項名詞。 |
| | 5 | (　) | 2.系列減七（5）：由 100 持續減 7，連續減五次答對，一個給一分。( 93. 86. 79. 72. 65)<br>如果個案不會計算，則請其執行倒著唸「台南火車站」或「家和萬事興」或 5 個不連續的數字。 |
| 三、記憶力(3) | 3 | (　) | 請個案說出剛剛所提的三項名詞。 |
| 四、語言(5) | 2<br>1 | (　)<br>(　) | 1.命名（2）：對筆及錶命名。例：(拿出手錶)這是什麼？<br>2.複誦（1）：請個案覆誦：「白紙真正寫黑字」或「有錢能使鬼推磨」。 |

| | | | |
|---|---|---|---|
| | 1<br>1 | (　　)<br>(　　) | 3.理解（1）：給個案看一張上面用大字印著「閉上眼睛」的紙，請個案讀出來，然後照做。<br>4.書寫造句（1）：請個案自己寫一句話。 |
| 五、口語理解及行為能力（3） | 3 | (　　) | 給個案一張空白無圖樣的紙，並且說「用你的右手拿紙（1），對摺（1），然後放在地板上(或再交給我)（1）」。一次說完這三個步驟之後再請個案執行。 |
| 六、建構力（1） | 1 | (　　) | 圖形抄繪(請個案將下列交疊的五角形描繪到一張白紙上)<br>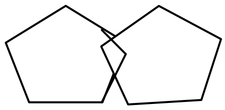 |

源自 Folstein, M., Folstein, S. E., & McHugh, P.(1975).Mini-mental state:a pactical method for grading the cognitive state:a practical method for grading the cognitive state if patient for the clinical.Journal of Psychiatric Research,12(3),189-198.

備註：有 11 個題目，30 個答案，達對一題得一分，答錯則不計分，滿分為 30 分，分數位於 24 -30 分為認知功能完整；18-23 分為輕度認知功能障礙，0-17 分為重度認知功能障礙。

## *Behavioral Intention*

Patient's Name: ______________________________ Date: ____________

| Items | 0 strongly disagree | 1 disagree | 2 neutral | 3 agree | 4 strongly agree |
|---|---|---|---|---|---|
| In your opinion, will you play and try different AVGs in the future? | | | | | |
| Do you think that AVGs can make you relaxed or vigorous? | | | | | |
| In your opinion, will you be interested in trying different types of AVGs? | | | | | |
| Do you think that AVGs can make you vigorous | | | | | |
| TOTAL | | | | | |

Note: Active video games (AVGs)

# Mini-Mental State Examination (MMSE)

Patient's Name: ________________________________________ Date: ____________

***Instructions:*** ***Ask the questions in the order listed. Score one point for each correct response within each question or activity.***

| | **Maximum Score** | **Patient's Score** | **Questions** |
|---|---|---|---|
| Orientation | 5 | | "What is the year? Season? Date? Day of the week? Month?" |
| | 5 | | "Where are we now: State? County? Town/city? Hospital? Floor?" |
| Attention and calculation | 3 | | The examiner names three unrelated objects (eg apple, table, penny), then asks the patient to name all three of them. Patient asked to repeat (1 point for each correct). |
| | 5 | | "I would like you to count backward from 100 by sevens." (93, 86, 79, 72, 65, …). Stop after five answers.<br>Alternative: " **(jia) (he) (wan) (shi) (xing)** backwards in order of characters" (**xing)(shi) (wan) (he) (jia**) |
| Memory | 3 | | "Earlier I told you the names of three things. Can you tell me what those were?" |
| Language | 2 | | Show the patient two simple objects, such as a wristwatch and a pencil, and ask the patient to name them. |
| | 1 | | "Repeat the phrase: **(bai)(zhi)(zhen)(zheng)(xie)(hei)(zi)** "On white paper, true words are written in black ink", or **(yo)(chyen)(nung)(shir)(gway)(tway)(maw)** "With money, you can make even ghosts push the millstone. " |
| | 1 | | "Please read this and do what it says." (Written instruction is "Close your eyes.") |
| | 1 | | "Make up and write a sentence about anything." (This sentence must contain a noun and a verb.) |
| Reasoning and problem solving | 3 | | "Take the paper in your right hand, fold it in half, and put it on the floor." (The examiner gives the patient a piece of blank paper.) |
| Executive function | 1 | | "Please copy this picture." (The examiner gives the patient a blank piece of paper and asks him/her to draw the symbol below. All 10 angles must be present and two must intersect.)<br>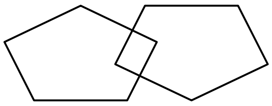 |
| | 30 | | TOTAL |

**Note:**

- **“家(jia)和（he）萬(wan)事(shi)興(xing)”：**

  (literally “Family harmony brings prosperity in everything.”)
- **“白（bai)紙（zhi)真（zhen)正（zheng)寫（xie)黑（hei)字（zi)”:**

  (literally “written in black on white paper,” and is equivalent to the English expression “in black and white.”)
- **“有(yo)錢(chyen)能(nung)使(shir)鬼(gway)推(tway)磨(maw):**

  (literally “With money, one can make even ghosts push the mill,” equivalent to “Money talks”).
